# Three‐dimensional geometric morphometric analysis of diaphragmatic dome motion in COPD patients

**DOI:** 10.1002/ar.70161

**Published:** 2026-02-11

**Authors:** José M. López‐Rey, Benoît Beyer, Elena García‐Vargas, Isabel Torres‐Sánchez, Francisco García‐Río, Markus Bastir

**Affiliations:** ^1^ Paleoanthropology Group, Department of Paleobiology Museo Nacional de Ciencias Naturales (MNCN‐CSIC) Madrid Spain; ^2^ Department of Biology, Faculty of Sciences Universidad Autónoma de Madrid (UAM) Madrid Spain; ^3^ Laboratory for Functional Anatomy, Faculty of Motor Sciences Université Libre de Bruxelles (ULB) Anderlecht Belgium; ^4^ Department of Anthropology and Human Genetics, Faculty of Sciences Univerzita Karlova Prague Czech Republic; ^5^ Pneumology Department, Hospital Universitario La Paz Instituto de Investigación Hospital Universitario La Paz (IdiPAZ) Madrid Spain; ^6^ Centro de Investigación Biomédica en Red de Enfermedades Respiratorias Instituto de Salud Carlos III Madrid Spain; ^7^ Department of Medicine, Faculty of Medicine Universidad Autónoma de Madrid Madrid Spain

**Keywords:** bronchitis, COPD, diaphragm, emphysema, geometric morphometrics, hyperinflation, lung

## Abstract

Diaphragmatic dysfunction is a hallmark of chronic obstructive pulmonary disease (COPD), especially in emphysema, where hyperinflation alters diaphragm geometry and impairs inspiratory mechanics. However, quantitative three‐dimensional (3D) assessments of diaphragmatic dome shape and motion across COPD phenotypes are limited. This study applied 3D geometric morphometrics (3DGM) to characterize diaphragmatic kinematics and curvature in COPD patients compared to controls. We prospectively enrolled 126 adults: 40 COPD patients (17 bronchitis‐predominant, 23 emphysema‐predominant) and 86 asymptomatic controls. All underwent high‐resolution computed tomography (CT) at functional residual capacity (FRC) and total lung capacity (TLC). Diaphragmatic form was quantified using landmark and semilandmark configurations. Curvature was estimated by fitting circular arcs to the domes. Statistical comparisons included Procrustes MANOVA, principal component analysis (PCA), and non‐parametric tests. Centroid size (CS) was significantly larger in COPD, reflecting dome flattening at both lung volumes. Inspiratory size increment (ΔCS) was reduced in emphysema, particularly in females. PCA showed diminished shape change between FRC and TLC in COPD, with overlapping configurations, in contrast to distinct phases in controls. PC2 highlighted flatter domes in COPD. Curvature analysis revealed significantly larger radii in COPD, especially at TLC. In COPD, the right dome was flatter than the left, whereas controls showed the opposite. The right dome retained greater curvature change, especially in emphysema. COPD patients, particularly those with emphysema and females, exhibit diaphragmatic flattening and reduced inspiratory deformation. Asymmetrical dome behavior, with preserved right dome mobility, may reflect compensatory adaptation. 3DGM offers a valuable tool for respiratory phenotyping in COPD.

## 1 INTRODUCTION

Chronic obstructive pulmonary disease (COPD) is a progressive and heterogeneous respiratory disorder, primarily associated with tobacco smoking, characterized by persistent and partially irreversible airflow limitation. This limitation arises from an abnormal inflammatory response to noxious inhalants, leading to bronchiolar obstruction, mucous hypersecretion, and alveolar destruction, the latter of which defines emphysema. Emphysema results in abnormal enlargement of distal airspaces and consequent air trapping, ultimately causing static and dynamic lung hyperinflation—an increase in residual lung volume due to expiratory airflow limitation (Gagnon et al., 2014; MacNee, 2006).

Lung hyperinflation has profound implications for the diaphragm. It shortens its cranio‐caudal length and flattens its domes, reducing its ability to generate pressure differentials during respiration (Cassart et al., 1997; García‐Río, 2005; Gauthier et al., 1994; Similowski et al., 1991). These biomechanical alterations increase the cost of breathing, contributing to respiratory muscle fatigue (Roussos, 1985) and oxygen‐demand‐induced remodeling of diaphragmatic fibers (Ottenheijm et al., 2008). Such effects may be more pronounced in emphysematous patients compared to those with predominant bronchitis phenotype, where air trapping is typically less severe (Gagnon et al., 2014; MacNee, 2006).

Sex‐related differences in COPD severity have also been reported, with some studies suggesting that females experience more pronounced disease manifestations (Sørheim et al., 2010), potentially due to smaller airway calibers (Hopkins & Harms, 2004) and reduced alveolar proliferation (Thurlbeck, 1982). This raises the possibility of differential diaphragmatic involvement by sex; however, this hypothesis remains untested.

While the structural and functional consequences of COPD on the diaphragm have been explored (Cassart et al., 1997; McKenzie et al., 2009; Salito et al., 2015), few studies have quantitatively assessed the three‐dimensional (3D) geometry of the diaphragmatic domes. To date, no research has employed 3D geometric morphometrics (3DGM) for this purpose. 3DGM enables rigorous quantification of shape based on Cartesian 3D coordinates of anatomical landmarks and semilandmarks—points distributed along curves or surfaces that acquire geometric homology through a sliding algorithm (Gunz et al., 2005; Gunz & Mitteroecker, 2013; Mitteroecker & Gunz, 2009). This method is especially suited for analyzing the complex curvature and surface topology of the diaphragm.

The present study aims to apply 3DGM to investigate inspiratory kinematic changes in the size and shape of the diaphragmatic domes in patients with COPD—specifically those with bronchitic or emphysematous phenotypes—compared to asymptomatic controls. We test the following hypothesis: (1) Inspiratory size and shape changes of the diaphragmatic domes are greater in asymptomatic individuals than in COPD patients; (2) Inspiratory kinematic changes are more pronounced in bronchitic patients than in those with emphysema; and (3) Diaphragmatic kinematic alterations during inspiration are more severe in female COPD patients than in male patients.

## 2 MATERIALS AND METHODS

### 2.1 Study subjects

We enrolled 126 adults: 40 COPD patients and 86 asymptomatic controls without airflow limitation. Participants were prospectively recruited from Hospital Universitario La Paz (Madrid, Spain), Hôpital Universitaire Erasme (Brussels, Belgium), and the Centro Nacional de Aceleradores (Seville, Spain) in collaboration with Univerzita Karlova (Prague, Czech Republic) and the Museo Nacional de Ciencias Naturales (MNCN‐CSIC, Madrid, Spain).

COPD patients met the following inclusion criteria: age ≥40 years, clinical diagnosis of COPD with post‐bronchodilator FEV_1_/FVC <0.7 and below the lower limit of normal, FEV_1_ <70% predicted, and ≥10 pack‐years of tobacco exposure (current or former smokers). All received optimized treatment per clinical guidelines, with no medication changes in the 8 weeks prior. Exclusion criteria included: ≥2 moderate or ≥1 severe exacerbations in the past year; systemic corticosteroids in the past 3 months; current long‐term oxygen therapy or mechanical ventilation; or any acute respiratory infection or exacerbation in the past 2 months. Further exclusions were history or suspicion of asthma, interstitial lung disease, cystic fibrosis, thoracic or pleural disorders, malignancy, or any neuromuscular, osteoarticular, or cognitive condition interfering with procedures or measurements.

Controls were never‐smokers with no history of respiratory or cardiovascular disease, no current symptoms (per ECSC questionnaire), and normal spirometry confirming no airflow limitation. All participants gave written informed consent. The study was approved by institutional ethics committees (HULP‐PI‐513; Erasme P2005/021; IRB Univerzita Karlova 2024/03) and complied with the Declaration of Helsinki (Goodyear et al., 2007).

### 2.2 Procedures

All participants underwent a standardized protocol including clinical examination, pulmonary function testing, and thoracic CT imaging. Pulmonary function tests followed ERS/ATS guidelines and included spirometry, lung volumes by plethysmography, and DLCO assessment (Bhakta et al., 2023; Graham et al., 2017, 2019). Reference values were based on GLI equations (Hall et al., 2021; Quanjer et al., 2012; Stanojevic et al., 2017).

CT scans were performed without contrast using Siemens Somatom or Biograph mCT systems with standardized parameters: slice width 0.3 mm, reconstruction increment 0.5 mm, collimation 16 × 0.75 mm, pitch 0.8, slice collimation 0.6 mm, 120 kVp, 160 mAs, and rotation time 0.6 s. During spiral CT from apex to base, participants received standardized coaching for breath‐holding at two lung volumes: TLC (deep inspiration) and FRC (end‐tidal expiration). High‐resolution images (B31f, B35f) were reconstructed in DICOM format for 3D modeling.

COPD patients were classified as bronchitis‐ or emphysema‐predominant using clinical features, pulmonary function, and CT findings (Miravitlles et al., 2014). Emphysema was defined by characteristic low‐attenuation areas on CT and hyperinflation markers (elevated RV, reduced IC, increased RV/TLC). Chronic bronchitis was defined by productive cough lasting ≥3 months in ≥2 consecutive years, without other causes or radiological evidence of emphysema.

### 2.3 3D morphometric analysis of diaphragmatic motion

3Dlung models were segmented from DICOM CT scans using 3D Slicer v5.6.2 (Fedorov et al., 2012) via the “Segment Editor” and “Grayscale Model Maker” modules. Resulting meshes were refined in MeshLab v2023.12 (Cignoni et al., 2008) to remove artifacts, smooth boundaries, and fill minor gaps.

Geometry of the upper diaphragm was quantified using 3DGM in Viewbox v4.1 (dHAL, Kifissia), applying a subset of 161 coordinates (4 landmarks/57 curve semilandmarks/100 surface semilandmarks) extracted from the template developed by Torres‐Tamayo et al. (2018) for quantifying lung form. Supplementary Figure S1 and Supplementary Table S1 detail the spatial distribution of the landmarks and semilandmarks used in this study. Diaphragmatic dome landmarks and semilandmarks were analyzed after generalized procrustes analysis (GPA) to remove size, position, and orientation effects (Mitteroecker & Gunz, 2009).

Shape variation was explored through principal component analysis (PCA) in shape space (Mitteroecker & Gunz, 2009), with group‐specific convex hulls and displacement vectors showing mean shape change from functional residual capacity (FRC) to total lung capacity (TLC). PCA plots using the most informative PCs (PC1 and PC2) were produced for all grouping factor combinations (lung volume, sex, clinical condition); only the most informative (Figure 1) appears in the main text, with others in Supplementary Figures S2–S6. Shape changes along the axes of Figure 1 were visualized by warping them onto a 3D diaphragm model segmented from a randomly selected control individual at FRC.

**FIGURE 1:**
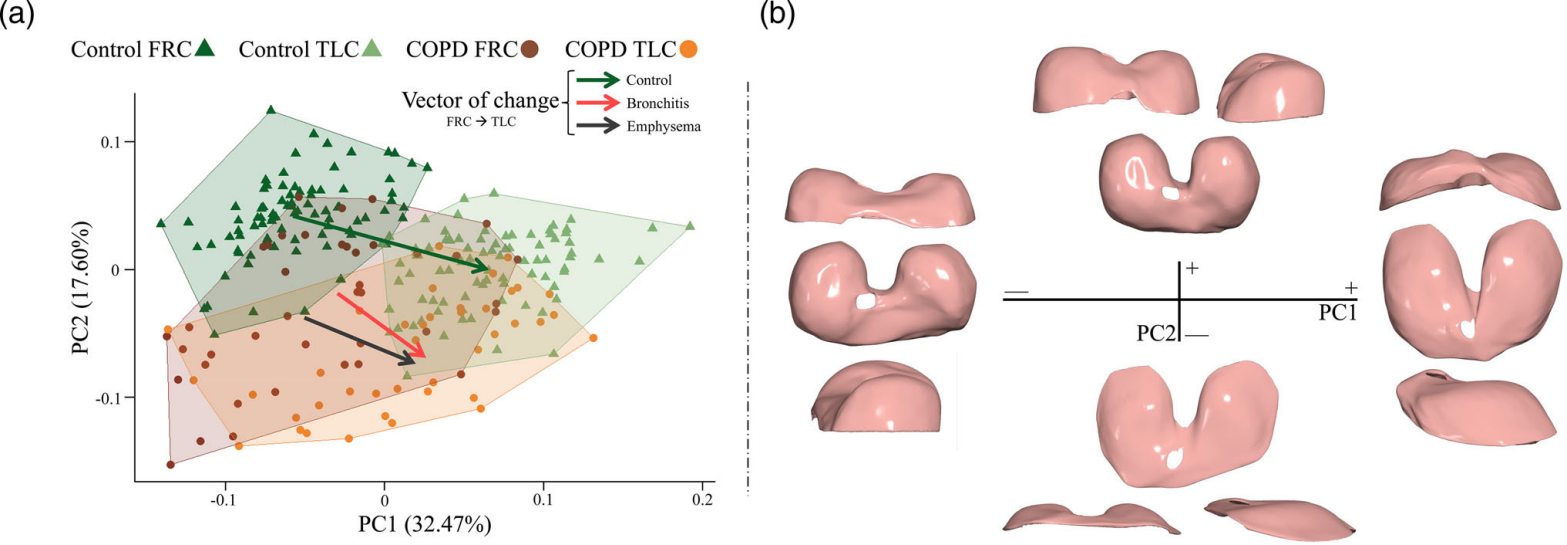
(a) Scatterplot showing the distribution of the principal component (PC) 1 and (PC2) scores. The sample is grouped according to lung volume (FRC, TLC) and condition (controls, COPD). (b) Visualizations of shape variation of the diaphragmatic domes along the axes.

Diaphragmatic size was assessed via centroid size (CS; Mitteroecker & Gunz, 2009) for both FRC and TLC states, and ΔCS between volume states was used to quantify inspiratory kinematics. Dome flatness was estimated by fitting a circular arc to each dome and calculating the radius as a curvature proxy (Table 2, Figure 2).

**FIGURE 2:**
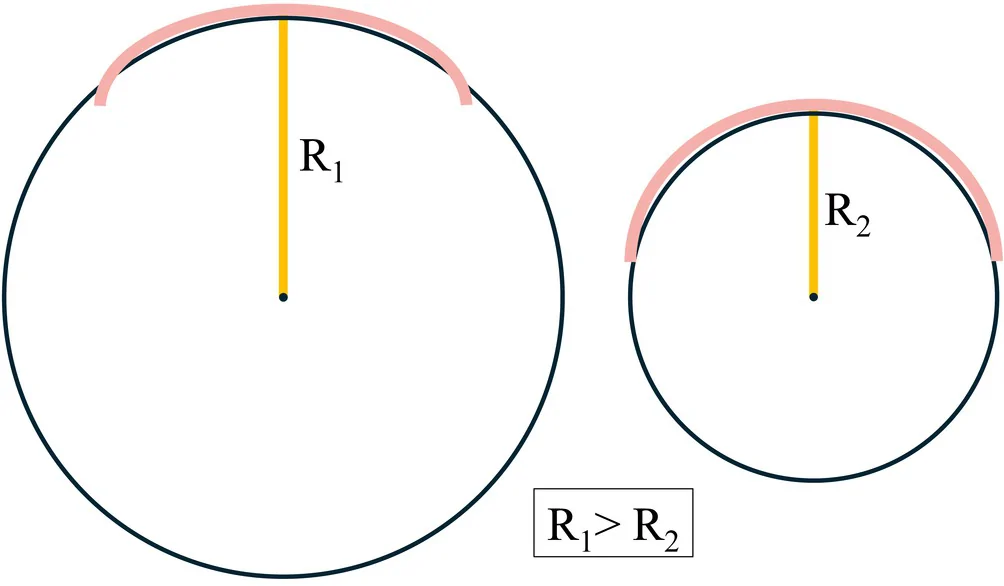
Schematic representation of how fitting a circle into a flatter diaphragmatic dome yields a larger radius (R).

### 2.4 Statistical analysis

Differences in diaphragmatic size (CS and ΔCS) and shape (PC scores) were compared across groups using non‐parametric statistical methods. Kruskal–Wallis tests were employed to assess global differences, followed by Dunn–Bonferroni post hoc tests for pairwise comparisons. Shape variation among categories defined by lung volume, sex, and clinical condition was evaluated using Procrustes multivariate analysis of variance (Procrustes MANOVA). Dome flatness measurements (curvature radii) were compared between groups using the Wilcoxon rank‐sum test and within individuals (left vs. right domes) using the Wilcoxon signed‐rank test.

All statistical analyses were performed in RStudio (version 2023.12.1‐402; R Core Team, 2023) using the following packages: dplyr (v1.1.2; Wickham et al., 2023), geomorph (v4.09; Adams et al., 2024), ggplot2 (v3.3.3; Wickham, 2016), Morpho (v2.10; Schlager, 2017), Rvcg (v0.22.1; Schlager, 2017), pracma (v2.2.4; Borchers, 2023), dunn.test (v1.3.6; Dinno, 2024), htmlwidgets (v1.6.2; Vaidyanathan et al., 2025), and base stats (v4.2.3; R Core Team, 2023). The complete analysis script is available in the Supplementary Online Material.

## 3 RESULTS

CS was significantly larger in COPD patients than in controls, and in males compared to females (Table 1). Dunn‐Bonferroni post hoc tests showed significant differences (*p* <0.05) between control females (FRC and TLC) and all males. Inspiratory change in CS (ΔCS) was greater in controls than in emphysema patients, with minimal differences between controls and bronchitis patients. The smallest ΔCS occurred in females with emphysema, though overall ΔCS differences were not significant (Kruskal–Wallis, *p* >0.05).

**TABLE 1:** Centroid size (CS) at FRC and TLC and inspiratory increment (ΔCS) in control subjects and COPD patients, stratified by clinical phenotype and sex^a^.

Group	CS at FRC	CS at TLC	ΔCS (TLC‐FRC)
Control subjects (*n* = 86)			
Females (*n* = 44)	1028.6 ± 106.7	1070.6 ± 121.6	42.0 ± 42.1
Males (*n* = 42)	1150.2 ± 148.7	1204.0 ± 176.8	53.8 ± 63.8
COPD patients (*n* = 40)			
Bronchitis (*n* = 17)			
Females (*n* = 7)	1144.6 ± 100.4	1177.3 ± 89.3	32.7 ± 29.5
Males (*n* = 10)	1281.9 ± 82.5	1346.2 ± 67.7	64.3 ± 31.4
Emphysema (*n* = 23)				
Females (*n* = 7)	1143.7 ± 106.8	1157.2 ± 97.9	13.5 ± 28.7	
Males (*n* = 16)	1267.7 ± 91.3	1304.7 ± 94.7	36.9 ± 63.8	

Abbreviations: CS, centroid size; FRC, functional residual capacity; TLC, total lung capacity.

^a^ Values are mean ± standard deviation.

Procrustes MANOVA revealed significant effects of lung volume (FRC vs. TLC), clinical group (controls, bronchitis, emphysema), and sex interaction on diaphragmatic shape (*p* <0.05), but post hoc comparisons between specific groups were not significant (*p* >0.05). Given the limited impact of sex on shape variation (Supplementary Figures S2–S6), PCA focused on lung volume and clinical group. PC1 (32.78%) separated FRC (negative) from TLC (positive); PC2 (17.40%) distinguished flatter (negative) vs. more curved domes (positive). Although PC3 explains 14.20% of the total shape variation, it was not considered in further analyses because the distribution of coordinates did not provide meaningful information regarding the hypotheses tested (positive: narrower aortic hiatus; negative: wider aortic hiatus). The remaining PCs were also not considered, as each explained less than 5% of the total shape variation.

The PCA plot (PC1‐PC2, Figure 1) showed that, compared to controls, COPD patients had (1) more overlap between FRC and TLC shapes and (2) flatter domes. PC score analysis confirmed significant differences (*p* <0.05, Dunn–Bonferroni), except between control‐FRC and COPD‐FRC (PC1), and control‐TLC and COPD‐FRC (PC2). While controls and emphysema patients followed similar vectors, bronchitis patients showed a more inclined trajectory, indicating altered deformation.

Diaphragmatic dome radii (Table 2) were generally larger (flatter domes) in COPD patients, with significant differences (*p* <0.05) in most comparisons—except the left dome in females with bronchitis at TLC, females with emphysema at FRC, and males with emphysema at TLC. Males had significantly larger radii only in the left dome of controls (FRC and TLC). Radii were greater at TLC versus FRC in all controls, the right dome of bronchitis females, and both domes in emphysema patients. Bilateral comparison showed flatter right domes in COPD and flatter left domes in controls; significant left–right differences appeared only in emphysema females at TLC.

**TABLE 2:** Estimated dome curvature (radius in mm) by condition, sex, lung volume phase, and side^a^.

Group	Radius at FRC		Radius at TLC	
	Left	Right	Left	Right
Control subjects				
Females	76.0 ± 14.9	79.0 ± 18.3	101.2 ± 21.2	100.3 ± 24.8
Males	86.8 ± 21.8	86.1 ± 29.3	119.5 ± 36.2	115.8 ± 49.5
COPD patients				
Bronchitis				
Females	103.2 ± 32.6	113.1 ± 33.5	141.0 ± 61.3	146.2 ± 61.3
Males	128.4 ± 37.2	140.9 ± 25.6	159.6 ± 48.0	195.7 ± 70.3
Emphysema				
Females	97.4 ± 32.5	116.6 ± 27.2	124.6 ± 35.6	159.3 ± 27.9
Males	114.7 ± 42.3	135.6 ± 38.3	141.1 ± 51.9	190.6 ± 92.9

Abbreviations: CS, centroid size; FRC, functional residual capacity; TLC, total lung capacity.

^a^ Values are mean ± standard deviation.

## 4 DISCUSSION

Our findings reveal that pathological condition exerts a significant influence on diaphragmatic dome size, as indicated by the larger CS observed in patients with COPD—both bronchitis and emphysema‐predominant—compared to asymptomatic individuals. This enlargement of CS at FRC and TLC is consistent with previous reports and reflects diaphragmatic flattening as a consequence of lung hyperinflation (Cassart et al., 1997; Salito et al., 2015). The geometric implication is a wider and less domed diaphragm, which may compromise mechanical efficiency by altering the muscle's ability to generate pressure.

At the muscular level, chronic hyperinflation shortens the diaphragm fibers, leading to a flattening of the domes [Correction added after first online publication on 02 April 2026. The previous sentence has been revised in this version.] (Cassart et al., 1997; Gauthier et al., 1994; Ottenheijm et al., 2008; Similowski et al., 1991). This altered geometry places the muscle fibers at a suboptimal length for force generation, reducing contractile efficiency and making the diaphragm more prone to fatigue (Ottenheijm et al., 2008; Roussos, 1985; Similowski et al., 1991). Additionally, the wider, less curved shape limits the piston‐like movement of the diaphragm during inspiration, further compromising its mechanical effectiveness (Cassart et al., 1997; García‐Río, 2005; Gauthier et al., 1994).

In this context, the reduced ΔCS observed in emphysema patients, particularly females, is noteworthy. A smaller inspiratory increase in CS suggests limited diaphragmatic dome expansion during inspiration, likely due to pre‐existing air trapping and increased lung volume at baseline. This finding aligns with the pathophysiology of emphysema, where destruction of alveolar walls and loss of elastic recoil lead to marked hyperinflation and reduced inspiratory capacity (Gagnon et al., 2014; MacNee, 2006). By contrast, patients with bronchitis showed ΔCS values more comparable to controls, suggesting that despite airway inflammation and mucous hypersecretion, the mechanical potential for diaphragmatic dome movement remains more preserved.

The observed sex differences, with more pronounced functional limitation in females with emphysema, may be explained by anatomical and physiological traits. Women have narrower airways relative to lung size (Hopkins & Harms, 2004) and a lower capacity for alveolar multiplication (Thurlbeck, 1982), which could exacerbate the effects of hyperinflation and limit diaphragmatic excursion.

Shape analysis through Procrustes MANOVA confirmed significant effects of lung volume, disease condition, and their interaction with sex, although post hoc pairwise comparisons did not reach statistical significance—likely due to correction for multiple comparisons or limited statistical power (Huberty & Lowman, 2000; Steyn Jr & Ellis, 2009). Importantly, sex alone did not emerge as a strong determinant of shape variation, underscoring that the dominant morphological drivers are respiratory phase and pathological status.

PCA provided further insight into these shape dynamics. Along PC1, which captured the transition from FRC to TLC, COPD patients exhibited considerable overlap between respiratory phases, whereas asymptomatic individuals showed clear separation, indicating greater shape change during inspiration. This reduced diaphragmatic adaptability in COPD is consistent with findings from recent imaging studies (Cao et al., 2022; Chen et al., 2022). PC2, meanwhile, reflected curvature variation, with more negative values in COPD patients denoting flatter domes. These changes likely indicate both structural remodeling and functional limitations associated with chronic inflammation and air trapping.

Vector analysis of mean shape change between lung volumes further confirmed that asymptomatic individuals experience greater diaphragmatic deformation, highlighting a preserved ability to modulate dome curvature in response to respiratory demands. Among COPD phenotypes, bronchitic patients showed the smallest vector magnitude, suggesting the lowest overall shape change. Interestingly, their dome shape at FRC was closer to controls than that of emphysematous patients, likely reflecting the absence of severe hyperinflation. However, at TLC, flattening appeared similarly pronounced across both phenotypes, supporting the notion that end‐inspiratory constraints may be a shared endpoint of COPD pathology.

The assessment of diaphragmatic flatness using curvature radii provided additional mechanistic insight. Larger radii were found in COPD patients compared to controls, reinforcing the observation of flatter domes and reduced mechanical advantage (Cassart et al., 1997; McKenzie et al., 2009; Salito et al., 2015). According to the law of Laplace, flatter diaphragmatic curvature translates to a lower capacity for pressure generation per unit of muscular tension (Gauthier et al., 1994), which could contribute to the ventilatory inefficiency observed in these patients. Sex‐based differences in dome curvature were also observed, with males displaying flatter domes, consistent with previous reports of anatomical and functional dimorphism in respiratory musculature (LoMauro & Aliverti, 2018).

Moreover, radii were consistently larger at TLC than at FRC across all groups, but this difference was statistically significant only in controls and selectively in the right dome of female patients with bronchitis and in both domes in emphysema patients. These findings suggest that, although diaphragmatic curvature changes during breathing, it is globally reduced in COPD (Cao et al., 2022; Chen et al., 2022). In addition, the right dome retains a degree of mobility, potentially linked to asymmetrical anatomic constraints. Bilateral comparisons between domes revealed an interesting pattern: in controls, the left dome was generally flatter, likely due to the anatomical presence of the heart limiting vertical excursion, whereas the liver supports and shapes the right dome (Standring, 2020). In contrast, in COPD patients, the right dome became flatter, suggesting altered dynamics possibly due to diminished abdominal support or postural changes secondary to hyperinflation. This reversal in dome asymmetry, especially when more pronounced in emphysema, could have functional consequences for breathing mechanics and may partly explain heterogeneity in clinical presentation or treatment response (Chen et al., 2022).

An additional factor that may influence diaphragmatic morphology is body size. While all our results are presented in absolute terms, it is possible that individual differences in body mass, particularly in the context of obesity, a common comorbidity in COPD (Putcha et al., 2015) could act as an additional anatomical constraint on the diaphragm, similar to the effects of the heart and liver. Including body size as a covariate could potentially modify the interpretation of diaphragmatic excursion and dome curvature, as larger individuals may exhibit different relative movements compared to smaller ones. Although analyzing these effects is beyond the scope of the current study, we highlight this as an interesting avenue for future research, which could provide further insight into inter‐individual variability in diaphragm mechanics and its functional consequences.

## 5 CONCLUSIONS

This study demonstrates that 3D geometric morphometrics is a powerful tool for quantifying diaphragmatic shape and motion in COPD. Our findings show that diaphragmatic domes in patients with bronchitis and emphysema are significantly flatter than those of asymptomatic individuals, and that their capacity for shape change during inspiration is markedly reduced —particularly in patients with emphysema and in females. The right dome appears to retain slightly more mobility than the left in COPD, in contrast to the pattern observed in controls. These structural and functional alterations in the diaphragm may underlie part of the ventilatory inefficiency in COPD and could inform future studies on respiratory muscle rehabilitation or personalized interventions based on phenotype and sex.

## AUTHOR CONTRIBUTIONS

**José M. López‐Rey:** Conceptualization; formal analysis; investigation; methodology; software; visualization; writing – original draft. **Benoît Beyer:** Writing – review and editing; validation; supervision; software; resources; methodology; investigation; formal analysis; conceptualization; data curation. **Elena García‐Vargas:** Data curation; resources; writing – review and editing. **Isabel Torres‐Sánchez:** Writing – review and editing; resources; data curation. **Francisco García‐Río:** Writing – review and editing; validation; supervision; resources; data curation. **Markus Bastir:** Conceptualization; data curation; formal analysis; funding acquisition; investigation; methodology; project administration; resources; software; supervision; validation; writing – review and editing.

## FUNDING INFORMATION

This research was supported by grant PRE2021‐097584 to J.M.L.‐R. and grant PID2020‐115854GB‐I00 to M.B., funded by MCIN/AEI/10.13039/501100011033 of the Spanish Ministry of Science and Innovation and the European Union.

## CONFLICT OF INTEREST STATEMENT

None of the authors and acknowledged researchers state any conflict of interest.

## Supporting information


**Data S1** Supplementary Online Material.

## Data Availability

Landmark data that support the findings of this study are available from the corresponding author to any researcher for purposes of reproducing or extending the analyses. Image data cannot be shared for reasons related to institutional agreements.
