# Supplementary material for: Three‐dimensional geometric morphometric analysis of diaphragmatic dome motion in COPD patients
Source: Anat Rec (Hoboken). 2026 Feb 11;309(12):3660–8. doi: 10.1002/ar.70161 (PMC13749314; doi:10.1002/ar.70161)
Supplement: Supplementary file 1 [file AR-309-3660-s001.docx]

**SUPPLEMENTARY ONLINE MATERIAL**

**Title:** Three-dimensional geometric morphometric analysis of diaphragmatic dome motion in COPD patients

**Supplementary Table S1.** Anatomical position of 4 landmarks (lm), 57 curve semilandmarks (Curve sml) and 100 surface semilandmarks (Surface sml) on the base of the lungs.

| **Number** | **Type** | **Anatomical position** |
| --- | --- | --- |
| 1 | lm | Apex middle lobe (Right) |
| 2 | lm | Lingula of left lung (Left) |
| 3 | lm | Lower lobe medial point (Right) |
| 4 | lm | Lower lobe medial point (Left) |
| 5 to 27 | Curve slm | Inferior margin of lung (Right) |
| 28 to 35 | Curve slm | Apex middle lobe (Right) – Lower lobe medial point curve (Right) |
| 36 to 53 | Curve slm | Inferior margin of lung (Left) |
| 54 to 61 | Curve slm | Apex middle lobe (Left) – Lower lobe medial point curve (Left) |
| 62 to 111 | Surface slm | Diaphragmatic surface (Right) |
| 112 to 161 | Surface slm | Diaphragmatic surface (Left) |

**Supplementary Figure S1.** Template of digitization. The morphology of the diaphragmatic domes was described using these landmarks (red), curve semilandmarks (green) and surface semilandmarks (orange and blue). Their anatomical position is indicated in Supplementary Table S1.

**Supplementary Figure S2.** Scatterplot showing the distribution of the principal component (PC) 1 and (PC2) scores. The sample is grouped according to lung volume and condition.

**Supplementary Figure S3.** Scatterplot showing the distribution of the principal component (PC) 1 and (PC2) scores. Only control individuals grouped by sex are represented.

**Supplementary Figure S4.** Scatterplot showing the distribution of the principal component (PC) 1 and (PC2) scores. Only COPD patients grouped by sex are represented.

**Supplementary Figure S5.** Scatterplot showing the distribution of the principal component (PC) 1 and (PC2) scores. Only females grouped by condition are represented.

**Supplementary Figure S6.** Scatterplot showing the distribution of the principal component (PC) 1 and (PC2) scores. Only males grouped by condition are represented

```
R SCRIPT
#Packages
library(dplyr)
library(dunn.test)
library(geomorph)
library(ggplot2)
library(Morpho)
library(rgl)
library(Rvcg)
library(pracma)
library(stats)
options(max.print=999999999)
getwd()


#A. RESLIDING
#1. Import the original dataset and select landmarks corresponding to the diaphragm
diaphragms <- read.morphologika("Definitive_dataset_252ind_2phases_161lmks.txt")

#2. Perform a GPA in form space (Morpho)
diaphragms_gpa <- procSym(diaphragms, CSinit = FALSE, scale = FALSE)

#3. Define landmarks (fix) and semilandmarks (curve)
{Fix <- c(1:4)
  Curve1 <- c(1,5:27,3)
  Curve2 <- c(1,28:35,3)
  Curve3 <- c(2,36:53,4)
  Curve4 <- c(2,54:61,4)
  Curves <- list(Curve1,Curve2,Curve3,Curve4)
  Surface <- c(1:161)[-c(Fix,Curve1,Curve2,Curve3,Curve4)]}

#4.2. Display the resliding
relaxarray <- diaphragms
for (i in 1:252) {
  print(i)
  relaxarray[,,i] <-  relaxLM(diaphragms[,,i],diaphragms_gpa$mshape,SMvector=Fix,
                              deselect=TRUE,outlines=Curves,surp=Surface,bending=TRUE)}

#4.3. Export the dataset with the estimated missing data
r2morphologika(relaxarray[,,1:252],"Definitive_dataset_252ind_2phases_161lmks_reslid.txt")


#B. GENERIC GEOMETRIC MORPHOMETRIC ANALYSIS
#B.1. SIZE
#CENTROID SIZE - RAW
diaphragms <- read.morphologika("Definitive_dataset_252ind_2phases_161lmks_reslid.txt")
diaphragms_gpa <- procSym(diaphragms)
CS_diaphragms <- diaphragms_gpa$size
groups_sex <- rep(c("Ctrl F EX", "Ctrl M EX", "Ctrl F IN", "Ctrl M IN",
                    "Emph F EX","Bronq F EX","Emph M EX","Bronq M EX",
                    "Emph F IN","Bronq F IN","Emph M IN","Bronq M IN"),
                  times = c(40, 46, 40, 46, 10, 8, 13, 9, 10, 8, 13, 9))

CS_dataframe <- data.frame(CentroidSize = CS_diaphragms,Group = groups_sex)

#Mean and SD
summary_CS <- aggregate(CentroidSize ~ Group, data = CS_dataframe,
                        FUN = function(x) c(Mean = mean(x), SD = sd(x)))
summary_CS

#Kruskal-Wallis + Dunn-Bonferroni
dunn_CS <- dunn.test(CS_diaphragms, groups_sex, method = "bonferroni")


#CENTROID SIZE - INCREMENT FRC -> TLC
diaphragms <- read.morphologika("Definitive_dataset_252ind_2phases_161lmks_reslid.txt")
diaphragms_gpa <- procSym(diaphragms)
CS_diaphragms <- diaphragms_gpa$size
groups_sex <- rep(c("Ctrl F EX", "Ctrl M EX", "Ctrl F IN", "Ctrl M IN",
                    "Emph F EX","Bronq F EX","Emph M EX","Bronq M EX",
                    "Emph F IN","Bronq F IN","Emph M IN","Bronq M IN"),
                  times = c(40, 46, 40, 46, 10, 8, 13, 9, 10, 8, 13, 9))
CS_dataframe <- data.frame(CentroidSize = CS_diaphragms, Group = groups_sex)
CS_dataframe$Condition <- ifelse(grepl("IN", CS_dataframe$Group), "IN", "EX")
CS_dataframe$GroupType <- gsub(" (IN|EX)", "", CS_dataframe$Group)
df_IN <- CS_dataframe %>% filter(Condition == "IN") %>% select(GroupType, CentroidSize)
df_EX <- CS_dataframe %>% filter(Condition == "EX") %>% select(GroupType, CentroidSize)
increments_individual <- data.frame(
  GroupType = df_IN$GroupType,
  Increment = df_IN$CentroidSize - df_EX$CentroidSize
)

#Mean + SD
increments_summary <- increments_individual %>%
  group_by(GroupType) %>%
  summarise(Mean = mean(Increment), SD = sd(Increment))

#Kruskal-Wallis + Dunn-Bonferroni
dunn_increment <- dunn.test(increments_individual$Increment,
                            increments_individual$GroupType,
                            method = "bonferroni")
print(increments_individual)
print(increments_summary)
print(dunn_increment)


#B.2. SHAPE
#GENERAL DIFFERENCES - PROCRUSTES MANOVA
#1. Import the dataset, peform a GPA (geomorph) and create groups according to sex
diaphragms <- read.morphologika("Definitive_dataset_252ind_2phases_161lmks_reslid.txt")
diaphragms_gpa <- gpagen(diaphragms)
condition <- rep(c("Control","COPD"), times=c(172,80))
condition <- rep(c("Control","Emphysema","Bronchitis","Emphysema","Bronchitis","Emphysema","Bronchitis","Emphysema","Bronchitis"), times=c(172,10, 8, 13, 9, 10, 8, 13, 9))
phase <- rep(c("EX","IN","EX","IN"), times=c(86,86,40,40))
sex <- rep(c("Females", "Males", "Females", "Males","Females", "Males", "Females", "Males"), times = c(40, 46, 40, 46, 18, 22, 18, 22))

#2. Create a geomorph dataframe
data_frame_proc_coords <- geomorph.data.frame(Shape = diaphragms_gpa$coords,
                                              Sex = sex,
                                              Condition = condition,
                                              Phase = phase)

#3. Perform the pairwise Procrustes MANOVA
proc_MANOVA <- procD.lm(Shape ~ Sex * Condition * Phase, data = data_frame_proc_coords)
summary(proc_MANOVA)
pairwise_results <- pairwise(proc_MANOVA, groups = interaction(data_frame_proc_coords$Sex,
                                                               data_frame_proc_coords$Condition,
                                                               data_frame_proc_coords$Phase))
pairwise_results
summary(pairwise_results)


#SPECIFIC DIFFERENCES - PCA SHAPE SPACE + VECTORS OF CHANGE
#1. Import the dataset, perform a GPA and create groups + dataframe
diaphragms <- read.morphologika("Definitive_dataset_252ind_2phases_161lmks_reslid.txt")
diaphragms_gpa <- procSym(diaphragms)
PC_scores <- diaphragms_gpa$PCscores
PC1 <- PC_scores[,1]
PC2 <- PC_scores[,2]
groups_generic <- rep(c("Ctrl EX", "Ctrl IN","COPD EX", "COPD IN"),
                      times = c(86, 86, 40, 40))
colours <- c("tomato4", "#F2862C","#09622A", "#81B274")
shapes <- c("Ctrl EX" = 17, "Ctrl IN" = 17, "COPD EX" = 16, "COPD IN" = 16)
data <- data.frame(PC1, PC2, PC3, groups_generic)

#2. Scatterplot
get_hull <- function(df) {
  df[chull(df$PC1, df$PC2), ]
}
hulls <- do.call(rbind, lapply(split(data, data$groups_generic), get_hull))

mean_ctrl_ex <- colMeans(data[data$groups_generic == "Ctrl EX", c("PC1", "PC2", "PC3")])
mean_ctrl_in <- colMeans(data[data$groups_generic == "Ctrl IN", c("PC1", "PC2", "PC3")])
mean_copd_ex <- colMeans(data[data$groups_generic == "COPD EX", c("PC1", "PC2", "PC3")])
mean_copd_in <- colMeans(data[data$groups_generic == "COPD IN", c("PC1", "PC2", "PC3")])

scatterplot_PC1_PC2 <- ggplot(data, aes(x = PC1, y = PC2, color = groups_generic, fill = groups_generic, shape = groups_generic)) +
  geom_point(size = 5) +
  geom_polygon(data = hulls, aes(x = PC1, y = PC2, group = groups_generic), alpha = 0.2) + # Convex hulls
  scale_color_manual(values = colours) +
  scale_fill_manual(values = colours) +
  scale_shape_manual(values = shapes) +
  theme_minimal() +
  theme(panel.grid = element_blank(),
        axis.line = element_line(color = "black"),
        axis.ticks = element_line(color = "black"),
        axis.text = element_text(size = 14)) +

  geom_segment(aes(x = mean_ctrl_ex[1], y = mean_ctrl_ex[2], xend = mean_ctrl_in[1], yend = mean_ctrl_in[2]),
               color = "#256D3D", size = 1.2) +
  geom_segment(aes(x = mean_copd_ex[1], y = mean_copd_ex[2], xend = mean_copd_in[1], yend = mean_copd_in[2]),
               color = "tomato4", size = 1.2)
print(scatterplot_PC1_PC2)

#Kruskal-Wallis + Dunn-Bonferroni
dunn_PC1 <- dunn.test(PC1, groups_generic, method = "bonferroni")
dunn_PC1
dunn_PC2 <- dunn.test(PC2, groups_generic, method = "bonferroni")
dunn_PC2

#NOTE: When we wanted to select specific individuals in the PCA, the code was
#e.g. CONTROL MALES VS CONTROL FEMALES
diaphragms <- read.morphologika("Definitive_dataset_252ind_2phases_161lmks_reslid.txt")
diaphragms_gpa <- procSym(diaphragms)
PC_scores <- diaphragms_gpa$PCscores
PC1 <- PC_scores[,1]
PC2 <- PC_scores[,2]
PC3 <- PC_scores[,3]
groups_sex <- rep(c("Control Females EX", "Control Males EX", "Control Females IN", "Control Males IN",
                    "COPD Females EX", "COPD Males EX", "COPD Females IN", "COPD Males IN"),
                  times = c(40, 46, 40, 46, 18, 22, 18, 22))
data <- data.frame(PC1, PC2, PC3, groups_sex)

selected_groups <- c("Control Females EX", "Control Males EX", "Control Females IN", "Control Males IN")
data_filtered <- subset(data, groups_sex %in% selected_groups)
shapes <- c("Control Females EX" = 17, "Control Males EX" = 16, "Control Females IN" = 17, "Control Males IN" = 16)
colours <- c("Control Females EX" = "pink4", "Control Males EX" = "steelblue4",
             "Control Females IN" = "pink", "Control Males IN" = "skyblue1")

get_hull <- function(df) {
  df[chull(df$PC1, df$PC2), ]
}

hulls_filtered <- do.call(rbind, lapply(split(data_filtered, data_filtered$groups_sex), get_hull))

scatterplot_PC1_PC2_filtered <- ggplot(data_filtered, aes(x = PC1, y = PC2, color = groups_sex, fill = groups_sex, shape = groups_sex)) +
  geom_point(size = 5) +
  geom_polygon(data = hulls_filtered, aes(x = PC1, y = PC2, group = groups_sex), alpha = 0.2) +
  scale_color_manual(values = colours) +
  scale_fill_manual(values = colours) +
  scale_shape_manual(values = shapes) +
  theme_minimal() +
  theme(panel.grid = element_blank(),
        axis.line = element_line(color = "black"),
        axis.ticks = element_line(color = "black"),
        axis.text = element_text(size = 14))
print(scatterplot_PC1_PC2_filtered)


#3. Visualize the extremes of each PC by a morphing
#Import the reference mesh, its correspondent coordinates and perform a GPA (geomorph)
diaphragms <- read.morphologika("Definitive_dataset_252ind_2phases_161lmks_reslid.txt")
mesh <- vcgImport("Diaphragm 2783237 simp proc Geom.ply",updateNormals = TRUE, readcolor = FALSE, clean = TRUE,silent = FALSE)
mesh_coordinates <- read.morphologika("DIA_2783237_EX.txt")
diaphragms_pca <- gm.prcomp(diaphragms_gpa$rotated)

#Perform the morphing and export the resulting meshes to the shape changes at the PC extremes
mesh_PC1neg <- tps3d(mesh,mesh_coordinates[,,1],diaphragms_pca$shapes$shapes.comp1$min)
mesh2ply(mesh_PC1neg, "PC1_neg.ply")
mesh_PC1pos <- tps3d(mesh,mesh_coordinates[,,1],diaphragms_pca$shapes$shapes.comp1$max)
mesh2ply(mesh_PC1pos, "PC1_pos.ply")
mesh_PC2neg <- tps3d(mesh,mesh_coordinates[,,1],diaphragms_pca$shapes$shapes.comp2$min)
mesh2ply(mesh_PC2neg, "PC2_neg.ply")
mesh_PC2pos <- tps3d(mesh,mesh_coordinates[,,1],diaphragms_pca$shapes$shapes.comp2$max)
mesh2ply(mesh_PC2pos, "PC2_pos.ply")


#C. RADII
#1. Split the dataset in left-right
diaphragms <- read.morphologika("Definitive_dataset_252ind_2phases_161lmks_reslid.txt")
L_dome <- diaphragms[c(2,4,36:61,112:161),,]
R_dome <- diaphragms[c(1,3,5:35,62:111),,]

#2. Split the obtained datasets according to lung volumes
L_dome_EX <- L_dome[,,c(1:86,173:212)]
L_dome_IN <- L_dome[,,c(87:172,213:252)]

R_dome_EX <- R_dome[,,c(1:86,173:212)]
R_dome_IN <- R_dome[,,c(87:172,213:252)]


#NOTE: x - mediolateral; y - anteroposterior; z - vertical

#3. Filter landmarks from a specific region in the X axis
L_dome_FRC_sagital_1 <- L_dome_EX[, , 1]
x_min <- min(L_dome_FRC_sagital_1[, 1])
x_max <- max(L_dome_FRC_sagital_1[, 1])
x_mid <- (x_min + x_max) / 2
tolerance <- 20

#4.Then we will filter those landmarks that are inside the calculated range
selected_landmarks <- L_dome_FRC_sagital_1[
  L_dome_FRC_sagital_1[, 1] >= (x_mid - tolerance) &
    L_dome_FRC_sagital_1[, 1] <= (x_mid + tolerance),
]

#5. Now remove the Y coordinate to have these landmarks in sagital view
selected_landmarks_sagital <- selected_landmarks[, c(2, 3)]

#6. Perform circle fitting on the selected landmarks
fit_circle <- circlefit(selected_landmarks_sagital[, 1], selected_landmarks_sagital[, 2])
#Circle parameters
center_x_sagital <- fit_circle[1]
center_z_sagital <- fit_circle[2]
radius_sagital <- fit_circle[3]

#6. Set the parameters of the circle
theta <- seq(0, 2 * pi, length.out = 100)
circle_x_sagital <- center_x_sagital  + radius_sagital  * cos(theta)
circle_z_sagital <- center_z_sagital  + radius_sagital  * sin(theta)
x_range_sagital <- range(selected_landmarks_sagital[, 1], circle_x_sagital)
z_range_sagital <- range(selected_landmarks_sagital[, 2], circle_z_sagital)
padding_sagital <- radius_sagital * 0.2
axis_limits_sagital <- c(
  min(x_range_sagital) - padding_sagital, max(x_range_sagital) + padding_sagital,
  min(z_range_sagital) - padding_sagital, max(z_range_sagital) + padding_sagital
)
plot(selected_landmarks_sagital, main = "Peripheral landmarks and fitted circle", xlab = "Y", ylab = "Z", pch = 19, col = "black", asp = 1)
points(selected_landmarks_sagital, col = "red", pch = 19)
lines(circle_x_sagital, circle_z_sagital, col = "blue", lwd = 2)
points(center_x_sagital, center_z_sagital, col = "green", pch = 19)

#7. Plot the circle
plot(selected_landmarks_sagital,
     main = "Perfil frontal - Individuo 1",
     xlab = "X", ylab = "Z", pch = 19, col = "red",
     xlim = axis_limits_sagital[1:2], ylim = axis_limits_sagital[3:4], asp = 1)
points(center_x_sagital, center_z_sagital, col = "green", pch = 19)
lines(circle_x_sagital, circle_z_sagital, col = "blue", lwd = 2)


#8 3D representation
#8.1. First, we have to define the function to adjust the circles
circlefit <- function(x, y) {
  n <- length(x)
  A <- cbind(2 * x, 2 * y, rep(1, n))
  b <- x^2 + y^2
  sol <- solve(t(A) %*% A) %*% t(A) %*% b
  center_x <- sol[1]
  center_y <- sol[2]
  radius <- sqrt(sol[3] + center_x^2 + center_y^2)
  c(center_x, center_y, radius)
}

#8.2. Sagital circle
x_min <- min(L_dome_FRC_sagital_1[, 1])
x_max <- max(L_dome_FRC_sagital_1[, 1])
x_mid <- (x_min + x_max) / 2
tolerance <- 50

selected_landmarks_sagital <- L_dome_FRC_sagital_1[
  L_dome_FRC_sagital_1[, 1] >= (x_mid - tolerance) &
    L_dome_FRC_sagital_1[, 1] <= (x_mid + tolerance),
]

sagital_coords <- selected_landmarks_sagital[, c(2, 3)]  # Mantener Y y Z
fit_circle_sagital <- circlefit(sagital_coords[, 1], sagital_coords[, 2])

center_y_sagital <- fit_circle_sagital[1]
center_z_sagital <- fit_circle_sagital[2]
radius_sagital <- fit_circle_sagital[3]

theta <- seq(0, 2 * pi, length.out = 100)
circle_y_sagital <- center_y_sagital + radius_sagital * cos(theta)
circle_z_sagital <- center_z_sagital + radius_sagital * sin(theta)
circle_x_sagital <- rep(mean(L_dome_FRC_sagital_1[, 1]), length(theta))

#8.3. 3D visualization of the landmarks + circle
#8.3.1. Define the ranges for each axis
x_range <- diff(range(L_dome_FRC_sagital_1[, 1]))
y_range <- diff(range(L_dome_FRC_sagital_1[, 2]))
z_range <- diff(range(L_dome_FRC_sagital_1[, 3]))

#8.3.2. Plot the landmarks on these axes
plot3d(L_dome_FRC_sagital_1[, 1], L_dome_FRC_sagital_1[, 2], L_dome_FRC_sagital_1[, 3],
       col = "black", size = 5, xlab = "X", ylab = "Y", zlab = "Z", main = "Circulos ajustados (sagital y coronal)",
       aspect = c(x_range, y_range, z_range))

#8.3.3. Plot the circle
lines3d(circle_x_sagital, circle_y_sagital, circle_z_sagital, col = "blue", lwd = 2)

#NOTE 1: Optionally, the centers of the circle can be added
points3d(mean(L_dome_FRC_sagital_1[, 1]), center_y_sagital, center_z_sagital, col = "blue", size = 10)

#NOTE 2: The process of calculating all radii can be authomatized by a loop
calculate_circle_fit <- function(data, phase, side, tolerance = 20) {
  results <- data.frame(
    Sample = integer(),
    Side = character(),
    Phase = character(),
    Center_X = numeric(),
    Center_Z = numeric(),
    Radius = numeric(),
    RMS_Error = numeric(),
    stringsAsFactors = FALSE
  )

  for (i in 1:dim(data)[3]) {
    sample_landmarks <- data[, , i]

    x_min <- min(sample_landmarks[, 1])
    x_max <- max(sample_landmarks[, 1])
    x_mid <- (x_min + x_max) / 2

    selected_landmarks <- sample_landmarks[
      sample_landmarks[, 1] >= (x_mid - tolerance) &
        sample_landmarks[, 1] <= (x_mid + tolerance),
    ]

    selected_landmarks_sagital <- selected_landmarks[, c(2, 3)]

    if (nrow(selected_landmarks_sagital) >= 3) {
      fit_circle <- circlefit(selected_landmarks_sagital[, 1], selected_landmarks_sagital[, 2])
      center_x <- fit_circle[1]
      center_z <- fit_circle[2]
      radius <- fit_circle[3]

      distances <- sqrt((selected_landmarks_sagital[, 1] - center_x)^2 +
                          (selected_landmarks_sagital[, 2] - center_z)^2)
      rms_error <- sqrt(mean((distances - radius)^2))

      results <- rbind(results, data.frame(
        Sample = i, Side = side, Phase = phase,
        Center_X = center_x, Center_Z = center_z,
        Radius = radius, RMS_Error = rms_error
      ))
    }
  }

  return(results)
}

results_L_EX <- calculate_circle_fit(L_dome_EX, "EX", "Left")
results_L_IN <- calculate_circle_fit(L_dome_IN, "IN", "Left")
results_R_EX <- calculate_circle_fit(R_dome_EX, "EX", "Right")
results_R_IN <- calculate_circle_fit(R_dome_IN, "IN", "Right")

final_results <- rbind(results_L_EX, results_L_IN, results_R_EX, results_R_IN)

head(final_results)

write.csv(final_results, "Diaphragm_Circle_Fitting_Results.csv", row.names = FALSE)

#9. Statistics
#9.1. Descriptive statistics of the sagital radius
bronq_F_EX_L_sag_radius <- c(80.24,92.64,95.74,166.15,95.01,66.21,137.90,91.83)
bronq_F_EX_R_sag_radius <- c(88.79,92.91,146.46,159.55,119.92,60.30,136.99,100.06)
bronq_M_EX_L_sag_radius <- c(144.71,118.63,171.64,190.59,94.82,110.10,137.75,115.79,71.36)
bronq_M_EX_R_sag_radius <- c(150.08,120.01,159.68,144.71,147.47,141.88,156.68,165.13,82.12)
bronq_F_IN_L_sag_radius <- c(102.26,111.36,119.15,206.01,140.89,76.11,112.14,259.91)
bronq_F_IN_R_sag_radius <- c(107.98,108.01,182.60,195.18,105.19,75.81,135.23,259.61)
bronq_M_IN_L_sag_radius <- c(171.79,168.48,137.77,160.57,189.15,243.01,159.21,144.98,61.42)
bronq_M_IN_R_sag_radius <- c(322.00,108.13,110.71,174.51,261.63,215.49,143.45,204.30,221.47)
emphy_F_EX_L_sag_radius <- c(78.31,89.90,75.42,161.76,87.31,49.86,110.51,73.63,121.78,125.28)
emphy_F_EX_R_sag_radius <- c(101.18,103.93,100.63,130.90,90.76,160.96,98.51,85.59,152.88,140.64)
emphy_F_IN_L_sag_radius <- c(125.83,90.45,163.39,152.32,169.23,57.69,88.56,133.00,131.31,134.21)
emphy_F_IN_R_sag_radius <- c(186.81,138.41,212.93,128.69,125.99,158.53,139.63,170.43,177.76,153.38)
emphy_M_EX_L_sag_radius <- c(90.18,124.98, 128.06,80.05,104.96,113.49,87.49,86.51,80.40,112.06,241.43,105.78,135.17)
emphy_M_EX_R_sag_radius <- c(154.78,233.91,101.31,97.75,163.54,113.73,114.56,147.81,98.29,138.91,113.50,118.79,165.68)
emphy_M_IN_L_sag_radius <- c(72.80,83.71,52.89,130.03,194.05,177.35,140.00,92.01,202.17,134.30,183.50,169.33,200.39)
emphy_M_IN_R_sag_radius <- c(137.09,239.05,62.18,135.57,429.18,184.66,158.78,165.41,154.86,199.44,305.61,115.38,190.85)
control_F_EX_L_sag_radius <- c(52.15,62.39,49.55,62.14,65.55,57.74,67.76,82.74,72.17,75.65,88.07,79.56,79.14,96.48,84.48,
                               99.77,95.97,95.20, 79.28, 74.99, 72.50, 91.08, 92.01, 85.74, 53.39, 73.91, 71.79, 82.52,
                               48.70, 54.93, 52.16, 82.72, 95.70, 67.05, 93.26, 95.10, 80.20, 88.00, 70.34, 66.35)
control_F_EX_R_sag_radius <- c(61.42, 51.92, 58.13, 47.76, 58.64, 59.11, 49.88, 75.28, 85.48, 78.23, 88.07, 75.22, 98.37,
                               83.97, 106.88, 104.74, 80.85, 98.24, 81.07, 78.06, 86.65, 101.86, 107.05, 109.30, 111.19,
                               61.82, 70.86, 67.03, 69.82, 93.47, 53.77, 62.96, 71.58, 75.77, 68.28, 90.93, 112.89, 80.36,
                               60.49, 75.75, 82.13)
control_M_EX_L_sag_radius <- c(52.65, 61.09, 53.20, 49.53, 90.45, 62.83, 116.42, 101.17, 97.94, 72.86, 73.38, 78.83, 94.28,
                               101.80, 113.93, 101.63, 153.86, 126.93, 71.32, 85.43, 99.50, 96.16, 100.31, 114.49, 74.40,
                               94.61, 81.27, 87.59, 69.78, 73.50, 84.98, 92.50, 110.39, 51.72, 91.54, 108.34, 71.24, 86.91,
                               84.21, 94.09, 60.13, 54.31, 88.19, 94.12, 105.73, 62.66)
control_M_EX_R_sag_radius <- c(53.41, 56.63, 56.43, 50.28, 70.22, 54.82, 121.12, 106.59, 84.37, 61.49, 75.02, 100.94, 129.13,
                               91.94, 109.33, 116.83, 215.11, 116.01, 64.55, 90.48, 117.03, 138.87, 96.15, 120.35, 88.30, 70.93,
                               83.27, 69.88, 67.65, 65.52, 86.22, 69.55, 79.59, 68.48, 95.44, 93.01, 77.05, 67.34, 66.19, 73.11,
                               65.19, 67.37, 70.43, 71.45, 89.75, 81.48)
control_F_IN_L_sag_radius <- c(66.11, 83.45, 63.98, 86.60, 93.23, 60.63, 144.51, 127.10, 93.01, 80.88, 95.91, 93.39, 117.07, 128.12,
                               113.29, 124.24, 137.54, 96.86, 108.56, 107.80, 118.67, 114.99, 133.47, 107.36, 77.18, 92.67, 144.70,
                               104.93, 92.28, 85.67, 91.64, 86.37, 85.81, 97.76, 88.56, 119.97, 85.53, 95.91, 119.10, 84.58)
control_F_IN_R_sag_radius <- c(61.08, 73.84, 56.48, 80.19, 87.43, 53.53, 83.13, 101.56, 95.65, 98.37, 123.05, 125.24, 128.63, 132.09,
                               131.93, 110.80, 125.95, 109.27, 100.59, 96.84, 119.49, 132.66, 156.24, 123.95, 68.84, 97.64, 134.57,
                               97.94, 123.52, 76.76, 122.96, 71.75, 72.98, 87.37, 108.50, 100.54, 92.10, 74.84, 96.02, 76.98,90.1)
control_M_IN_L_sag_radius <- c(64.03, 82.47, 79.34, 64.92, 90.28, 89.62, 147.25, 138.95, 150.41, 119.02, 103.69, 111.41, 191.14,
                               202.80, 156.06, 115.14, 133.25, 163.06, 77.30, 102.24, 152.61, 226.36, 106.79, 118.70, 129.67, 111.97,
                               158.60, 92.31, 99.42, 77.99, 117.53, 166.85, 126.61, 108.26, 110.64, 126.95, 107.56, 91.77, 109.43, 94.56,
                               94.75, 96.85, 97.19, 113.69, 185.69, 92.91)
control_M_IN_R_sag_radius <- c(69.24, 61.19, 92.88, 60.57, 73.96, 73.63, 155.74, 137.20, 135.53, 86.30, 104.26, 105.89, 233.41, 123.64,
                               116.78, 131.46, 356.06, 153.23, 76.38, 109.88, 170.52, 198.99, 119.08, 141.83, 105.88, 97.18, 136.80, 95.06,
                               86.04, 106.36, 106.68, 109.12, 130.15, 109.21, 108.49, 116.40, 90.34, 94.32, 84.86, 69.45, 88.84, 85.61, 105.63,
                               111.32, 108.48, 94.28)

#Mean and standard deviation
mean(bronq_F_EX_L_sag_radius)
sd(bronq_F_EX_L_sag_radius)
mean(bronq_F_EX_R_sag_radius)
sd(bronq_F_EX_R_sag_radius)
mean(bronq_M_EX_L_sag_radius)
sd(bronq_M_EX_L_sag_radius)
mean(bronq_M_EX_R_sag_radius)
sd(bronq_M_EX_R_sag_radius)
mean(bronq_F_IN_L_sag_radius)
sd(bronq_F_IN_L_sag_radius)
mean(bronq_F_IN_R_sag_radius)
sd(bronq_F_IN_R_sag_radius)
mean(bronq_M_IN_L_sag_radius)
sd(bronq_M_IN_L_sag_radius)
mean(bronq_M_IN_R_sag_radius)
sd(bronq_M_IN_R_sag_radius)

mean(emphy_F_EX_L_sag_radius)
sd(emphy_F_EX_L_sag_radius)
mean(emphy_F_EX_R_sag_radius)
sd(emphy_F_EX_R_sag_radius)
mean(emphy_M_EX_L_sag_radius)
sd(emphy_M_EX_L_sag_radius)
mean(emphy_M_EX_R_sag_radius)
sd(emphy_M_EX_R_sag_radius)
mean(emphy_F_IN_L_sag_radius)
sd(emphy_F_IN_L_sag_radius)
mean(emphy_F_IN_R_sag_radius)
sd(emphy_F_IN_R_sag_radius)
mean(emphy_M_IN_L_sag_radius)
sd(emphy_M_IN_L_sag_radius)
mean(emphy_M_IN_R_sag_radius)
sd(emphy_M_IN_R_sag_radius)

mean(control_F_EX_L_sag_radius)
sd(control_F_EX_L_sag_radius)
mean(control_F_EX_R_sag_radius)
sd(control_F_EX_R_sag_radius)
mean(control_M_EX_L_sag_radius)
sd(control_M_EX_L_sag_radius)
mean(control_M_EX_R_sag_radius)
sd(control_M_EX_R_sag_radius)
mean(control_F_IN_L_sag_radius)
sd(control_F_IN_L_sag_radius)
mean(control_F_IN_R_sag_radius)
sd(control_F_IN_R_sag_radius)
mean(control_M_IN_L_sag_radius)
sd(control_M_IN_L_sag_radius)
mean(control_M_IN_R_sag_radius)
sd(control_M_IN_R_sag_radius)

#Pairwise comparison control-copd
wilcox.test(bronq_F_EX_L_sag_radius,control_F_EX_L_sag_radius)
wilcox.test(bronq_F_IN_L_sag_radius,control_F_IN_L_sag_radius)
wilcox.test(bronq_M_EX_L_sag_radius,control_M_EX_L_sag_radius)
wilcox.test(bronq_M_IN_L_sag_radius,control_M_IN_L_sag_radius)
wilcox.test(bronq_F_EX_R_sag_radius,control_F_EX_R_sag_radius)
wilcox.test(bronq_F_IN_R_sag_radius,control_F_IN_R_sag_radius)
wilcox.test(bronq_M_EX_R_sag_radius,control_M_EX_R_sag_radius)
wilcox.test(bronq_M_IN_R_sag_radius,control_M_IN_R_sag_radius)

wilcox.test(emphy_F_EX_L_sag_radius,control_F_EX_L_sag_radius)
wilcox.test(emphy_F_IN_L_sag_radius,control_F_IN_L_sag_radius)
wilcox.test(emphy_M_EX_L_sag_radius,control_M_EX_L_sag_radius)
wilcox.test(emphy_M_IN_L_sag_radius,control_M_IN_L_sag_radius)
wilcox.test(emphy_F_EX_R_sag_radius,control_F_EX_R_sag_radius)
wilcox.test(emphy_F_IN_R_sag_radius,control_F_IN_R_sag_radius)
wilcox.test(emphy_M_EX_R_sag_radius,control_M_EX_R_sag_radius)
wilcox.test(emphy_M_IN_R_sag_radius,control_M_IN_R_sag_radius)

#Pairwise comparison male-female
wilcox.test(bronq_F_EX_L_sag_radius,bronq_M_EX_L_sag_radius)
wilcox.test(bronq_F_IN_L_sag_radius,bronq_M_IN_L_sag_radius)
wilcox.test(bronq_F_EX_R_sag_radius,bronq_M_EX_R_sag_radius)
wilcox.test(bronq_F_IN_R_sag_radius,bronq_M_IN_R_sag_radius)

wilcox.test(emphy_F_EX_L_sag_radius,emphy_M_EX_L_sag_radius)
wilcox.test(emphy_F_IN_L_sag_radius,emphy_M_IN_L_sag_radius)
wilcox.test(emphy_F_EX_R_sag_radius,emphy_M_EX_R_sag_radius)
wilcox.test(emphy_F_IN_R_sag_radius,emphy_M_IN_R_sag_radius)

wilcox.test(control_F_EX_L_sag_radius,control_M_EX_L_sag_radius)
wilcox.test(control_F_IN_L_sag_radius,control_M_IN_L_sag_radius)
wilcox.test(control_F_EX_R_sag_radius,control_M_EX_R_sag_radius)
wilcox.test(control_F_IN_R_sag_radius,control_M_IN_R_sag_radius)

#Pairwise comparison FRC-TLC
wilcox.test(bronq_F_EX_L_sag_radius,bronq_F_IN_L_sag_radius,paired=TRUE)
wilcox.test(bronq_F_EX_R_sag_radius,bronq_F_IN_R_sag_radius,paired=TRUE)
wilcox.test(bronq_M_EX_L_sag_radius,bronq_M_IN_L_sag_radius,paired=TRUE)
wilcox.test(bronq_M_EX_R_sag_radius,bronq_M_IN_R_sag_radius,paired=TRUE)

wilcox.test(emphy_F_EX_L_sag_radius,emphy_F_IN_L_sag_radius,paired=TRUE)
wilcox.test(emphy_F_EX_R_sag_radius,emphy_F_IN_R_sag_radius,paired=TRUE)
wilcox.test(emphy_M_EX_L_sag_radius,emphy_M_IN_L_sag_radius,paired=TRUE)
wilcox.test(emphy_M_EX_R_sag_radius,emphy_M_IN_R_sag_radius,paired=TRUE)

wilcox.test(control_F_EX_L_sag_radius,control_F_IN_L_sag_radius,paired=TRUE)
wilcox.test(control_F_EX_R_sag_radius,control_F_IN_R_sag_radius,paired=TRUE)
wilcox.test(control_M_EX_L_sag_radius,control_M_IN_L_sag_radius,paired=TRUE)
wilcox.test(control_M_EX_R_sag_radius,control_M_IN_R_sag_radius,paired=TRUE)

#Pairwise comparison L-R
wilcox.test(bronq_F_EX_L_sag_radius,bronq_F_EX_R_sag_radius,paired=TRUE)
wilcox.test(bronq_M_EX_L_sag_radius,bronq_M_EX_R_sag_radius,paired=TRUE)
wilcox.test(bronq_F_IN_L_sag_radius,bronq_F_IN_R_sag_radius,paired=TRUE)
wilcox.test(bronq_M_IN_L_sag_radius,bronq_M_IN_R_sag_radius,paired=TRUE)

wilcox.test(emphy_F_EX_L_sag_radius,emphy_F_EX_R_sag_radius,paired=TRUE)
wilcox.test(emphy_M_EX_L_sag_radius,emphy_M_EX_R_sag_radius,paired=TRUE)
wilcox.test(emphy_F_IN_L_sag_radius,emphy_F_IN_R_sag_radius,paired=TRUE)
wilcox.test(emphy_M_IN_L_sag_radius,emphy_M_IN_R_sag_radius,paired=TRUE)

wilcox.test(control_F_EX_L_sag_radius,control_F_EX_R_sag_radius,paired=TRUE)
wilcox.test(control_M_EX_L_sag_radius,control_M_EX_R_sag_radius,paired=TRUE)
wilcox.test(control_F_IN_L_sag_radius,control_F_IN_R_sag_radius,paired=TRUE)
wilcox.test(control_M_IN_L_sag_radius,control_M_IN_R_sag_radius,paired=TRUE)
```
